# Metagenomics Analysis of the Microbial Consortium in Samples from Lake Xochimilco, a World Cultural Heritage Site

**DOI:** 10.3390/microorganisms13040835

**Published:** 2025-04-07

**Authors:** Alvaro de Obeso Fernández del Valle, Jorge Membrillo-Hernández

**Affiliations:** 1 Bioengineering Department, School of Engineering and Sciences, Tecnologico de Monterrey, Mexico City Campus, Calle del Puente 222, Col. Ejidos de Huipulco, Tlalpan, Mexico City 14380, Mexico; adeobeso@tec.mx; 2 Institute for the Future of Education, Tecnologico de Monterrey, Av. Eugenio Garza Sada 2501, Col. Tecnologico, Monterrey 64700, Mexico

**Keywords:** metagenomics, *16S* RNA, phylum, chinampa, SDG11, cultural heritage, biotechnology, higher education, sustainability

## Abstract

Since ancient times, the community of Xochimilco in the south of Mexico City has provided vegetables for the entire city. Today, Lake Xochimilco is listed as a UNESCO World Heritage Site because it is the last remaining bastion of Aztec culture and preserves the extraordinary ecological landscape of chinampas, a system of arable islands that has endured for over 1000 years. Here, we report on the microbiological communities currently existing in the lake. This is relevant since the water irrigates crops on the islands, known as chinampas. To achieve this, samples from the lake were collected at two different sites, and metagenomics analysis of the *16S* gene was conducted. The results indicate the presence of five dominant bacterial phyla: *Actinobacteria* (44.5%), *Proteobacteria* (22.5%), *Firmicutes* (13%), *Bacteroidota* (6%), and *Chloroflexi* (4.6%). The most abundant families were *Micrococcaceae*, *Intrasporangiaceae*, and *Rhodobacteraceae*. The results correlate with current anthropogenic activity, indicating a moderate problem associated with contamination. Our findings suggest that immediate actions and increased awareness are necessary to preserve this cultural and natural heritage site and to take steps to comply with Sustainable Development Goal 11 (Sustainable Cities and Communities). Furthermore, this is the first report to characterize microbial communities in the water of Lake Xochimilco using *16S* rRNA gene sequencing.

## 1. Introduction

Understanding the bacterial composition of Lake Xochimilco through metagenomics analysis is crucial for preserving its ecological balance and ensuring the safety of its multiple uses. As a UNESCO World Heritage site, Lake Xochimilco is renowned for its ancient chinampas—floating gardens that have sustained agriculture for centuries—and its vibrant tourism industry featuring traditional trajinera boat rides. The lake’s health has a direct impact on the productivity of these chinampas and the quality of the tourist experience. Metagenomics enables a comprehensive assessment of microbial communities, offering insights into water quality, nutrient cycles, and potential pathogenic threats. This knowledge is essential for developing effective management strategies to maintain the lake’s ecological integrity [1,2]

**Figure 1:**
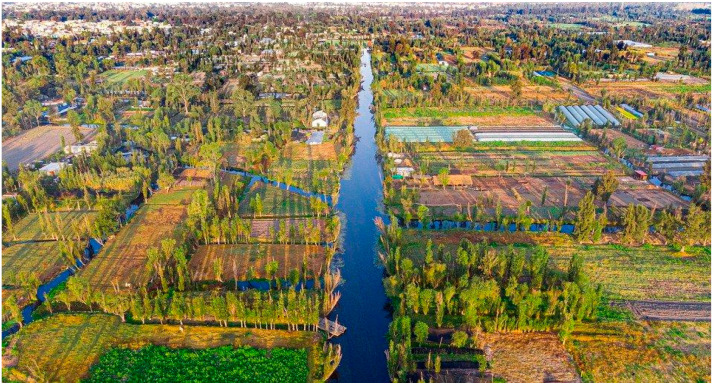
Aerial view of Xochimilco Lake and Chinampas.

The chinampas rely on the lake’s water for irrigation, making understanding its microbial content vital for agricultural sustainability. Metagenomics studies can identify beneficial bacteria that support plant growth and detect harmful microorganisms that may compromise crop health or pose risks to consumers. Farmers can implement informed practices to enhance soil fertility and crop yields by monitoring these microbial populations while minimizing health hazards (Figure 1). This approach not only preserves traditional farming methods but also promotes food security for the local community [2,3,4].

Tourism in Xochimilco is closely tied to the lake’s condition, as visitors are drawn to its unique ecosystem and rich cultural heritage. The presence of waterborne pathogens could deter tourism and harm the local economy. Metagenomics analysis enables the early detection of such threats, facilitating timely interventions to protect public health [5]. Moreover, understanding the microbial dynamics aids in maintaining the aesthetic and environmental quality of the waterways, ensuring a safe and enjoyable experience for tourists. Thus, metagenomics research is a proactive measure to sustain both the ecological and economic vitality of Lake Xochimilco.

**Figure 2:**
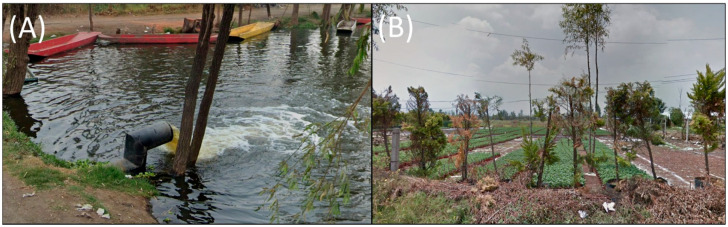
Images of Lake Xochimilco. (**A**) Effluent waters filling Xochimilco Lake. (**B**) Chinampa on the Lake. Images were obtained from Google Maps at coordinates (19.2606387, −99.0748693) and (19.2650673, −99.0733166).

Despite being a highly productive agricultural system, Lake Xochimilco faces numerous threats, including intensive farming practices, pesticide usage, pest infestations, soil erosion, the introduction of non-native species (both aquatic and terrestrial), regional climate changes, forest degradation, flooding, land subsidence, irregular human settlements, and rapid urbanization that contribute to soil and water contamination [3,6,7]. Additionally, the 187 km of canals are now maintained using water from a treatment plant (Figure 2), which is utilized for irrigating crops in the Chinampas region.

Some studies have been conducted to quantify certain polluting microorganisms in the waters of Lake Xochimilco [3,8,9,10]. Coliforms have been detected in samples of irrigation water, soil, and vegetables grown in chinampas [3]. The existence of microbial communities capable of adapting to the chinampas environment and making it highly productive was recently reviewed [10]. Microbiological analysis of the chinampas soil through pyro-sequencing was recently reported [10]. However, there is no recent data on microbiology available in the water of Xochimilco Lake.

Metagenomic studies on Lake Xochimilco provide valuable insights into its microbial diversity and ecological functions. However, aquatic ecosystems are highly dynamic, with microbial communities constantly changing due to environmental factors such as pollution, climate variability, and anthropogenic activities. Previous studies established a baseline of knowledge, but continuous monitoring is crucial for understanding how these ecosystems evolve [11]. New metagenomic studies can reveal shifts in microbial composition, the emergence of potential pathogens, and changes in key functional genes related to biogeochemical cycles. These insights are essential for developing conservation strategies, mitigating pollution, and preserving the unique biodiversity of this World Heritage Site. Furthermore, recent advances in sequencing technologies and bioinformatics enable a deeper and more precise characterization of microbial communities than previously possible. Given the ecological and cultural importance of Lake Xochimilco, updated metagenomic studies are necessary to inform evidence-based conservation efforts and policy decisions that can safeguard its sustainability for future generations.

Indeed, changes in microbial communities can serve as early indicators of lake pollution and ecosystem disturbances, as microorganisms respond rapidly to environmental changes. Shifts in microbial diversity, dominance of specific taxa, and alterations in functional groups, such as nitrogen-fixing or sulfate-reducing bacteria, can signal pollution before visible environmental degradation occurs. Metagenomic monitoring enables the real-time tracking of microbial diversity, allowing for the early detection of stressors. Since microbial imbalances may signal deteriorating water quality that affects aquatic organisms and human health, monitoring microbial communities in lakes like Xochimilco can help detect pollution early and support sustainable water management.

Additionally, monitoring specific microbial indicators is crucial for evaluating long-term sustainability in line with Sustainable Development Goal 11 (SDG 11)**.** Key indicators include *Escherichia coli* and *Enterococcus* spp. for fecal contamination; *Nitrosomonas* and *Nitrospira* for maintaining a balanced nutrient environment; and *Desulfovibrio* spp. for detecting anoxic conditions. Harmful cyanobacteria, such as *Microcystis*, signal nutrient pollution, while antibiotic-resistant bacteria and pathogens, including *Klebsiella pneumoniae*, *Legionella pneumophila*, and *Pseudomonas aeruginosa*, indicate risks of contamination. Regular metagenomic monitoring of these microbes supports sustainable water management and the conservation of ecosystems.

The area’s biodiversity is under serious threat. For instance, the native Axolotl salamander (*Ambystoma mexicanum*) is now endangered due to competition with invasive species, such as tilapia [12]. Furthermore, the current farmers, who are between 70 and 90 years old, show little interest in passing down their traditional knowledge to younger generations, posing a risk to the continuity of this unique ecosystem [13].

In addition, the 2030 Agenda of the United Nations Member States established the Sustainable Development Goals (SDGs) to promote knowledge generation. SDG 11 aims to make cities and human settlements inclusive, safe, resilient, and sustainable [14]. Specifically, target 11.4 of SDG 11 emphasizes the need to protect and preserve the world’s cultural and natural heritage [14]. In line with this goal, conducting an updated study on the microbial populations in Lake Xochimilco would enhance our understanding of the site’s sustainability.

## 2. Materials and Methods

### 2.1. Sample Collection

**Figure 3:**
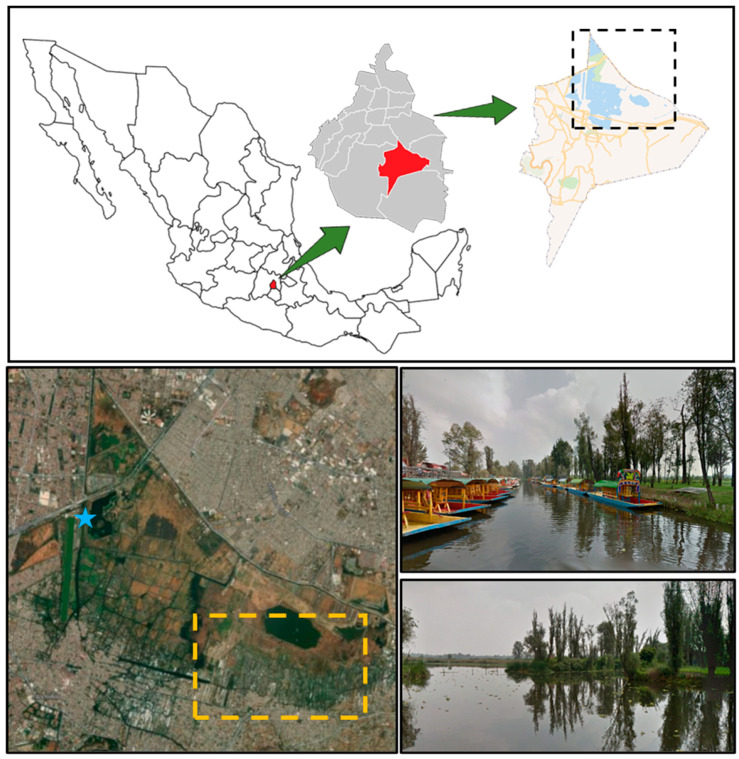
Sampling location. The blue star indicates the location of Cuemanco Pier (Google Maps Coordinates: 19.2874, −99.1019).

The sample collection locations were determined based on the results being helpful to the Xochimilco community; therefore, two samples were collected: one near the chinampas (cultivation sites) and the other at the piers (sites of high anthropogenic activity). Water samples were collected 1 m from the edge of the mainland at 9 a.m. on 28 July 2024, at the Cuemanco Pier (19.287914702063127°, −99.10241914029469°) in Xochimilco, Mexico City, Mexico (Figure 3). This site was chosen due to its proximity to the Chinampas zone. Two samples were taken at different locations using Erlenmeyer flasks. The water was filtered, and the paper filter was washed to collect all microorganisms. The samples were taken to the laboratory in a cooler at 4 °C for further processing.

### 2.2. Nucleic Acid Extraction

Samples were pretreated by freezing at −80 °C and crushed with mortar. Samples were then treated with acetone. After drying for several hours, a DNeasy PowerSoil Pro Kit (QIAGEN, Hilden, Germany) was used following the manufacturer’s instructions. Water samples are first passed through the beads provided. Then, several buffers are used to clean the sample from soil particles and other compounds, such as acidic molecules, while isolating the cells in the water. Cells are lysed, and the sample is run through a purification column. DNA is eluted and quantified after cleanup, and purity is assessed using a NanoDrop 2000C spectrophotometer (Thermo Scientific, Wilmington, DE, USA).

### 2.3. 16S rDNA Amplification and Data Analysis

Primers targeting the variable V3 and V4 regions of the *16S* rRNA gene, which included adapter regions compatible with the Nextera XT Index kit [15], were used to prepare the sequencing libraries according to the *16S* Metagenomic Sequencing Library Preparation protocol [15]. Negative controls (non-template controls) were included in each reaction. All libraries were quantified with Qubit dsDNA HS Assay Kit (Invitrogen, Carlsbad, CA, USA), library size was analyzed in a QSep 400 (BiOptic, New Taipei City, Taiwan), and sequencing was performed in a MiSeq using a MiSeq Reagent kit V3 (Illumina, San Diego, CA, USA) in a 301 bp pair-end reads configuration. Primers and adapters were removed from the demultiplexed sequence read, and data quality was assessed using the FastQC tool [16,17]. Sequences were trimmed using a Phred score threshold of 20 after quality control assessment. Amplicon sequence variants (ASV) were obtained using the DADA2 R package [18,19]. Fifteen nucleotides were removed from the reads’ start and truncated at 250 bases. Any reads mapping to the *phiX* genome containing an unassigned base or with an expected error < 2 were removed. After error learning and denoising, corresponding paired reads were merged, and chimeras were identified and removed using the consensus method of the “removeBimeraDenovo” function of DADA2. The SILVA nr99 v138.1 database [20] was used for the taxonomy assignment. ASV and taxonomy tables were merged into a phyloseq [21] object in R for analysis. Graphics were generated with the ggplot2 R package [22].

## 3. Results and Discussion

Conducting metagenomics analysis of the bacteria in Lake Xochimilco is crucial for preserving the ecological integrity and cultural significance of this UNESCO World Heritage site. As the lake supports traditional agricultural practices through the Chinampas system and sustains a thriving tourism industry, understanding its microbial composition is essential for ensuring water quality and public health. Metagenomics analysis provides a comprehensive overview of bacterial communities, including the detection of potentially harmful pathogens and antibiotic-resistant genes that could pose serious risks to human health and agricultural productivity. Moreover, this approach provides valuable insights into nutrient cycling and ecosystem functioning, which are essential for maintaining the delicate balance necessary for sustainable agriculture and environmental conservation. By establishing a baseline of microbial diversity and monitoring changes over time, this study can inform effective management practices to combat pollution, invasive species, and other environmental stressors. Ultimately, the findings contribute to the development of evidence-based policies that safeguard Lake Xochimilco’s unique ecosystem, support the livelihoods of local communities, and preserve its cultural heritage.

**Figure 4:**
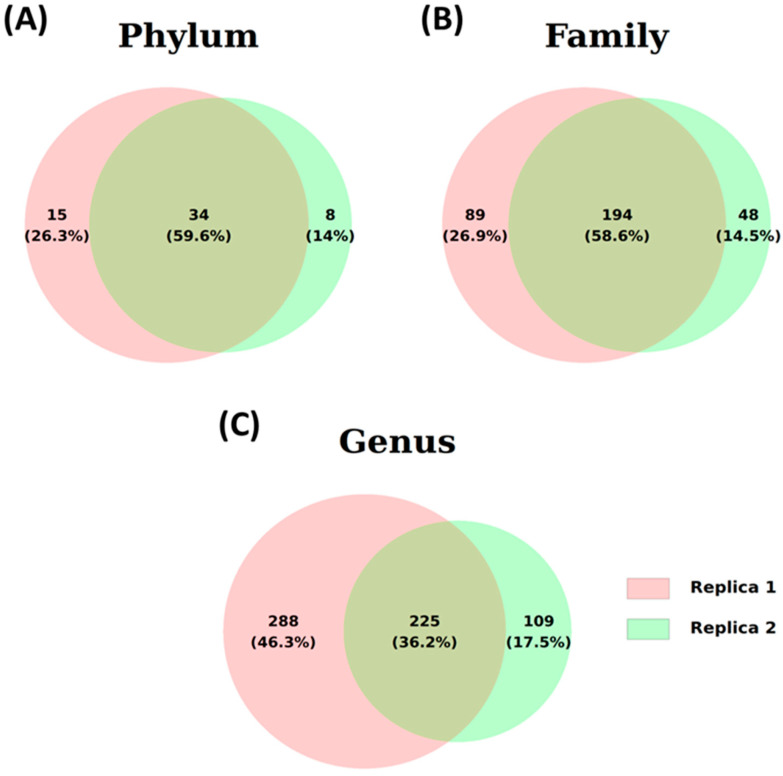
Illustrative Venn diagram of identified ASVs for two water samples (Samples 1 and 2) at the three taxonomic levels: (**A**) phylum, (**B**) family, and (**C**) genus.

Two different water samples from Lake Xochimildo were taken for examination. The sequences obtained through metagenomics, based on the *16S* rRNA gene, were analyzed using the Phylum, Family, and Genus programs. Figure 4 presents the results in Venn diagrams, illustrating the distribution of total sequences identified at each taxonomic level. At the Phylum level, 57 different phyla were counted. Both samples had 36 phyla in common, representing 59.6% of the total (Figure 4A). Of the 331 other families identified, the two samples shared 196 families (58.6%) (Figure 4B). At the Genus level, 622 genera were found, of which 225 genera (36.5%) were present in both samples (Figure 4C). The number of common sequences across different taxonomic levels strongly suggests consistency in the analyzed samples.

The high proportion of shared taxa suggests ecologically similar microbial communities influenced by common environmental factors. Unique taxa may indicate distinct ecological conditions, such as differences in nutrient availability or pollution levels. While shared taxa could reflect functional redundancy, additional data are needed to confirm environmental similarity.

Table 1 shows the 20 main phyla found in the two samples collected from the Xochimilco Lake water, their prevalence (number of ASVs found for each taxonomic group), and the percentage abundance of these phyla. Phyla with less than 1% abundance were incorporated into the same category (Phyla < 1 relative abundance). Based on the database, the unassigned (NA) category corresponds to ASVs that were counted but could not be assigned to a known phylum.

As observed, 15 phyla are present in both samples. Eight of them (*Gemmatimonadota*, *Bdellovibrionota*, *Armatimonadota*, *Calditrichota*, *Fibrobacterota*, *Campylobacterota*, *Halobacterota*, and *Nanoarcheota*) are only in one of them. The dominant phylum is *Actinobacteria*, with an abundance of 42 to 47%. *Proteobacteria* is the second most abundant phylum, accounting for 19 to 26% of the total abundance, followed by *Firmicutes*, *Bacteroidota*, and *Chloroflexi*, which comprise 7 to 19%, 4 to 8%, and 4 to 5%, respectively (Table 1). The average relative abundances corresponded to 93% of the biodiversity of the identified phyla. A very low percentage of sequences (less than 0.055%) were from phyla not assigned (NA).

Table 2 indicates the composition of the community at the family level. The families with the highest proportion are *Micrococcaceae*, *Intrasporangiaceae*, and *Rhodobacteraceae*, with abundances ranging from 12% to 32%, 5% to 12%, and 3% to 8%, respectively. Only 9 of the top 20 families were identified in both samples. There was a high ASV unassigned (NA) value, with an abundance of approximately 9–11%. Families with less than 1% abundance were clustered together.

*Actinobacteria*, *Proteobacteria*, *Chloroflexi*, and *Cyanobacteria* are phyla reported in other lakes naturally [23,24]. *Actinobacteria* are free-living Gram-positive microorganisms widely distributed in aquatic and terrestrial ecosystems [25]. *Proteobacteria* are physiologically diverse Gram-negative bacteria; many are free-living nitrogen-fixing bacteria but are also important pathogens of plants, animals, and humans [26]. *Chloroflexi* is an anoxygenic phototrophic phylum responsible for carbonate precipitation and detected in natural water bodies [27]. The presence of *Actinobacteria*, *Proteobacteria*, *Firmicutes*, and *Bacteroidota* phyla is also associated with anthropomorphic activities [28]. Human activities in the Xochimilco Lake are intense, and there are even open-air drains that are discharged into the lake without any control; a previous study indicated that soil samples and vegetables grown in chinampas had a high number of total coliforms and fecal coliforms (phylum *Proteobacteria*) compared to the maximum permissible limits for human consumption. Most coliforms were found in spinach, lettuce, and parsley [6]. In particular, coriander and lettuce crops exhibited high levels of fecal and mesophilic aerobic coliforms (*Enterobacter* genus) and *Salmonella typhi* [8].

Table 3 shows the most abundant genera found in the samples of Lake Xochimilco. *Kocuria* is the most abundant genus (10–32%) and is common in both samples. Two additional genera present in both samples were *Blastococcus* and *Sphingomonas*. However, 20–26% of the sequences could not be assigned. Most of the top 20 genera belong to the phyla *Actinobacteria* and *Proteobacteria*. *Actinobacteria*, *Proteobacteria*, *Chloroflexi*, and *Cyanobacteria* are phyla that have been reported in other natural lakes [23,24]. *Actinobacteria*, *Proteobacteria*, *Firmicutes*, and *Bacteroidota* phyla are associated with anthropomorphic activities [29]. As mentioned, previously total and fecal coliforms (genus *Enterobacter* and phylum *Proteobacteria*) were reported as pathogens exceeding the maximum permissible limits in vegetables [3], in the same way that *Streptococcus* (*Firmicutes* phylum), *Micrococcus* (*Actinobacteria* phylum), and non-fermenting bacteria, such as *Pseudomonas* and *Acetobacter* (*Proteobacteria* phylum), were identified in samples from the wastewater treatment plant used to feed the Lake [9].

It has also been possible to isolate bacteria of the type Streptococcus (*Firmicutes* phylum), Micrococcus (*Actinobacteria* phylum), and non-fermenting bacteria, such as Pseudomonas and Acetobacter (*Proteobacteria* phylum), in samples from the wastewater treatment plant, which today fills the Xochimilco Lake [9]. It is important to note that most of the phyla found in *Firmicutes*, *Bacteroidota,* and *Actinobacteria*, including more than 200 genera with *Mycoplasma*, *Bacillus,* and *Clostridium* in a more significant proportion, have been found in the human digestive tract as pathogens, most of them [28,30]. Families of *Proteobacteria* have been reported to include human pathogenic bacteria, such as the genus Enterobacter (including coliforms), as noted in the work of Rosas [6], as well as microorganisms carrying antibiotic resistance genes [31]. Our findings correlate with these anthropomorphic microbial communities, as the most abundant families identified in our samples were *Micrococcaceae* and *Intrasporangiaceae*. Furthermore, *Clostridiaceae* is present within the top 20 families. The distribution of *Micrococcaceae* (phylum *Actinobacteria*) is found in soil and aqueous environments, as well as on the skin of mammals, primarily *Kocuria* spp., with the potential to cause infections associated with human biomedical devices [32]. The higher abundance of *Intrasporangiaceae* (phylum *Actinobacteria*) has been linked to putative polyphosphate-accumulating organisms that are abundant in many large-scale bioremediation plants [32,33,34,35] Members of this family have been isolated from marine and lake sediments, soil, salt mines, mine waste, activated sludge, marine waters, and the upper stratosphere [34], as well as possibly opportunistic pathogens [36].

Previous DNA pyrosequencing analyses of microorganisms from chinampa soils identified the phyla *Proteobacteria*, *Chloroflexi*, *Firmicutes*, *Acidobacteriota*, *Bacteroidota*, *Actinobacteria*, *Chlorobi*, *Gemmatimonadetes*, *Nitrospirae*, and *Plantomycetota* [10]. Seven phyla and two genera of the most abundant microorganisms identified in our water samples coincide with those found in the soil, suggesting a dynamic interaction between soil and water in the chinampa zone. Common genera were *Sphingomonas* (phylum *Alphaproteobacteria*) and *Bacillus* (phylum *Firmicutes*). Trujillo-Cabrera et al. reported that the phyla *Proteobacteria*, *Actinobacteria*, and *Firmicutes* were identified in a soil analysis of chinampas [36]. Nitrogen content was a determining factor for the composition of the microbial community associated with the sulfur cycle in the rhizosphere of plants growing in chinampa soil [10].

When relative phyla abundances in Lake Xochimilco are compared to those from sequencing analysis of seven polluted urban lakes in India [29], a report of three lakes in Xian, China and a report on urban water systems in Bandar Sunway, Malaysia, *Proteobacteria*, *Bacteroidetes*, *Firmicutes*, and *Actinobacteria* are the dominant bacterial phyla (Table 4) [37,38]. Actinobacteria were ten times more abundant in our samples than in Indian lakes, followed by Proteobacteria, which were predominant in those polluted lakes and three times more abundant than in Lake Xochimilco. In the same way, the phylum *Bacteroidota* had relative abundances three times higher than those of Lake Xochimilco. The distribution of microbial phyla can provide an indication of the degree of contamination in the lakes. Although the physicochemical properties of the water were not analyzed, based on the results of the microbial ecosystem and a comparison with the literature [30,36], we can conclude that urban settlements have had a moderately negative impact on Lake Xochimilco [29,39]. Therefore, we recommend taking measures to preserve, treat, or implement solutions that reduce water contamination and ensure the quality of crops for human consumption. Future pathogen characterization, such as in the James River in 2015 [31] or Indian Lakes [29], could provide additional supporting information to expedite immediate actions. Undoubtedly, the actions based on these findings are crucial in preserving this valuable cultural heritage. “Omics” techniques are tools that can be used in this monitoring. Recent studies highlight its role in diagnosing, monitoring, and protecting historical and cultural assets [40,41]. Its usefulness in analyzing the effect of biodeterioration and environmental variables on these heritages has also been highlighted [36,38]. It is essential to note that microbial community compositions can vary significantly between urban lakes, influenced by land-use patterns, pollution levels, and water chemistry.

## 4. Conclusions

The presence of *Actinobacteria*, *Proteobacteria*, *Firmicutes*, *Bacteroidota*, and *Chloroflexi* in an urban water body suggests a complex and diverse microbial community influenced by natural and anthropogenic factors [44]. This indicates that the lake is moderately to highly likely to harbor pathogenic bacteria. Some phyla contain genera associated with waterborne diseases, antibiotic resistance, and fecal contamination.

1. *Proteobacteria* (High Possibility). Proteobacteria are highly versatile and encompass a wide range of pathogenic and non-pathogenic species. Their presence is familiar in urban water bodies due to their adaptability to diverse environmental conditions, including pollution. Depending on the specific genera present, they can be indicators of sewage contamination or nutrient enrichment. This phylum encompasses many known pathogens and is commonly found in contaminated water bodies. Potential pathogenic genera include the following: *Escherichia coli*, *Salmonella*, *Klebsiella*, *Pseudomonas*, *Vibrio*, and *Legionella*. They are widely found in sewage-impacted water and are associated with gastrointestinal infections, respiratory diseases, and skin infections [45].

2. *Actinobacteria* (Low to Moderate Possibility)**.** These bacteria are primarily decomposers, but some species are opportunistic pathogens. Potential pathogenic genera include the following: *Mycobacterium* (*M. avium*, *M. tuberculosis*). These bacteria can cause pulmonary infections and are often found in aerosolized water sources, such as lakes, showers, and cooling towers [46]. Actinobacteria play a crucial role in decomposing organic matter, thereby contributing to the cycling of essential nutrients. Their presence may indicate a natural, productive ecosystem, but high abundance could also be associated with organic pollution.

3. *Bacteroidota* (Moderate Possibility). They are also associated with fecal pollution and are indicators of organic matter decomposition. High levels might suggest contamination from wastewater or urban runoff. *Bacteroides* is frequently related to fecal contamination and an anaerobic environment, and is a potential pathogenic genus, along with *Prevotella*. While most are commensal gut bacteria, some species are opportunistic pathogens that can cause wound infections and sepsis [47].

4. *Chloroflexi* (Low Possibility). This is typically non-pathogenic and involved in biogeochemical cycling. It is found in sediments and wastewater but is not directly linked to human disease [48].

5. *Firmicutes* (Moderate Possibility). This group includes both harmless and pathogenic species, some of which are capable of forming spores. Potential pathogenic genera include *Clostridium* (*C. difficile*, *C. botulinum*), *Bacillus* (*B. cereus*, *B. anthracis*), and *Staphylococcus* (*S. aureus*). Some are associated with foodborne illness, wound infections, and toxin production [47]. Firmicutes are often linked to fecal contamination, as many species are found in the gut microbiota of humans and animals. Their presence could indicate sewage or agricultural runoff, potentially posing public health risks.

These findings suggest a dynamic microbial ecosystem influenced by urbanization, likely reflecting contamination sources, such as sewage, agricultural runoff, or industrial pollutants. The phyla identified in this manuscript may suggest that bacteria in Lake Xochimilco are associated with Chinampa agriculture, where fertilizers, wastewater irrigation, and decomposing plants introduce nutrient-cycling bacteria. Fecal contamination from livestock may introduce enterobacteria, which may stem from runoff. Monitoring these microbes is crucial for the sustainability of Chinampa and the protection of its water quality.

Further analysis, including the identification of specific genera and assessment of functional genes, would be necessary to better understand the water body’s health status, contamination sources, and potential risks to public health and the environment. Our results indicate that the microbiological quality of Xochimilco Lake water is poor, posing a risk to human consumption. To the best of our knowledge, this is the first study to characterize the microbial communities in the water of Lake Xochimilco and utilize metagenomic sequencing to assess the state of biodeterioration of this natural heritage.

Microbial metagenomics is vital for lake conservation policies. It provides insights into microbial diversity, ecosystem health, and pollution indicators, such as pathogenic bacteria and antibiotic-resistant genes. These data help design strategies for pollution control, waste management, and water quality monitoring. Additionally, metagenomics identifies beneficial microbes that support nutrient cycling and water purification, aiding in bioremediation efforts. Metagenomics, which tracks microbial biodiversity over time, informs adaptive policies aimed at mitigating the impacts of urbanization, tourism, and industrial activities. Integrating these findings into environmental regulations can promote sustainable land use and stricter wastewater treatment standards. Evidence-based policymaking using microbial data enhances lake resilience and ensures long-term conservation. It does not escape our attention that the population dynamics of the microorganisms present can vary according to the specific conditions of each season. Future studies will include annualized research to determine the dynamics of microbial populations present in Lake Xochimilco.

Functional metagenomic analyses can provide valuable information on the presence of bacteria with antibiotic-resistance genes; therefore, experiments are underway to determine this. Future research and long-term lake microbiome monitoring should focus on standardized sampling and the integration of multi-omics to track microbial shifts and their corresponding functional roles. Regular pathogen and antibiotic resistance surveillance is essential for assessing public health risks. Longitudinal studies should evaluate the impact of climate change and pollution on microbial communities. Machine learning can enhance predictive modeling for ecosystem changes while engaging policymakers and local communities to ensure the development of effective conservation strategies. Researchers can develop early warning systems and sustainable water management practices by combining these approaches.

## Figures and Tables

**Table 1:** Top 20 phyla for water samples from Xochimilco Lake.

Sample 1			Sample 2		
Phylum	Prevalence	Abundance (%)	Phylum	Prevalence	Abundance (%)
*Actinobacteria*	41,983	42.69	*Actinobacteria*	27,381	46.68
*Proteobacteria*	18,919	19.24	*Proteobacteria*	15,224	25.96
*Firmicutes*	16,750	17.03	*Bacteroidota*	4467	7.62
*Chloroflexi*	4755	4.84	*Chloroflexi*	2586	4.41
*Bacteroidota*	4430	4.50	*Firmicutes*	2093	3.57
*Patescibacteria*	2114	2.15	*Fusobacteriota*	1286	2.19
*Cyanobacteria*	2006	2.04	*Patescibacteria*	1186	2.02
*Plantomycetota*	1164	1.18	*Cyanobacteria*	1011	1.72
*Gemmatimonadota*	956	0.97	*Desulfobacterota*	655	1.12
*Verrucomicrobiota*	807	0.82	*Verrucomicrobiota*	566	0.97
*Acidobacteriota*	692	0.70	*Spirochaetota*	452	0.77
*Desulfobacterota*	648	0.66	*Deinococcota*	422	0.72
*Myxococcota*	648	0.66	*Acidobacteriota*	249	0.42
*Spirochaetota*	623	0.63	*Fibrobacterota*	197	0.34
*Bdellovibrionota*	435	0.44	*Plantomycetota*	176	0.30
*Deinococcota*	326	0.33	*Campylobacterota*	153	0.26
*Fusobacteriota*	227	0.23	*Myxococcota*	109	0.19
*Armatimonadota*	80	0.08	*Halobacterota*	78	0.13
*Calditrichota*	75	0.08	*Nanoarchaeota*	39	0.07
Unassigned	25	0.03	Unassigned	27	0.05

**Table 2:** Top 20 families for water samples of Xochimilco Lake.

Sample 1			Sample 2		
Family	Prevalence	Abundance (%)	Family	Prevalence	Abundance (%)
*Micrococcaceae*	12,505	12.72	*Micrococcaceae*	18,635	31.77
*Intrasporangiaceae*	11,322	11.51	*Rhodobacteraceae*	4608	7.86
*Bacillaceae*	6673	6.79	*Intrasporangiaceae*	3408	5.81
*Nocardioidaceae*	5006	5.09	*Azospirillaceae*	1602	2.73
*Rhodobacteraceae*	3742	3.81	*Rhodocyclaceae*	1480	2.52
*Planococcaceae*	3182	3.24	*Acetobacteraceae*	1372	2.34
*Geodermatophilaceae*	2758	2.80	*Leptotrichiaceae*	1286	2.19
*JG30-KF-CM45*	2113	2.15	*JG30-KF-CM45*	980	1.67
*Flavobacteriaceae*	1642	1.67	*Anaerolineaceae*	978	1.67
*Sphingomonadaceae*	1592	1.62	*Marinilabiliaceae*	799	1.36
*Cellulomonadaceae*	1495	1.52	*Propionibacteriaceae*	785	1.34
*Rhizobiaceae*	1103	1.12	*Sphingomonadaceae*	739	1.26
*Comamonadaceae*	1098	1.12	*Geodermatophilaceae*	707	1.21
*Clostridiaceae*	965	0.98	*Acidothiobacillaceae*	688	1.17
*Rhodocyclaceae*	942	0.96	*Prolixibacteraceae*	639	1.09
*Paenibacillaceae*	923	0.94	*Comamonadaceae*	455	0.78
*Xanthomonadaceae*	798	0.81	*Bacteroidota vadinHA17*	441	0.75
*Micromonosporaceae*	795	0.81	*Dermabacteraceae*	440	0.75
*Anaerolineaceae*	792	0.81	*Aeromonadaceae*	435	0.74
Unassigned	10,782	10.96	Unassigned	5758	9.82

**Table 3:** Top 20 genera for water samples from Xochimilco Lake.

Sample 1			Sample 2		
Genus	Prevalence	Abundance (%)	Genus	Prevalence	Abundance (%)
*Kocuria* *	10,627	10.81	*Kocuria* *	18,464	31.48
*Ornithinimicrobium*	5597	5.69	*Paracoccus*	4181	7.13
*Bacillus*	3995	4.06	*Arsenicicoccus*	3080	5.25
*Nocardioides*	3017	3.07	*Skermanella*	1602	2.73
*Serinicoccus*	2388	2.43	*Roseomonas*	902	1.54
*Blastococcus* *	2227	2.26	*[Cytophaga] xylanolytica group*	710	1.21
*Paracoccus*	1999	2.03	*KCM-B-112*	688	1.17
*Marmoricola*	1603	1.63	*Blastococcus* *	594	1.01
*Ornithinicoccus*	1394	1.42	*Luteococcus*	579	0.99
*Cellulomonas*	1105	1.12	*Dechloromonas*	472	0.80
*Skermanella*	779	0.79	*Brachybacterium*	438	0.75
*Planococcus*	718	0.73	*Deinococcus*	407	0.69
*Antarcticibacterium*	713	0.73	*Aeromonas*	391	0.67
*Pseudarthrobacter*	707	0.72	*Belnapia*	374	0.64
*Amaricoccus*	698	0.71	*Sphingomonas* *	374	0.64
*Sporosarcina*	646	0.66	*Paludibacter*	336	0.57
*Clostridium sensu stricto 1*	624	0.63	*Chitinivorax*	326	0.56
*Sphingomonas* *	624	0.63	*Thauera*	294	0.50
*Paenibacillus*	597	0.61	*Rubellimicrobium*	288	0.49
Unassigned	25,534	25.97	Unassigned	12,232	20.86

* Common taxa in both samples.

**Table 4:** The top microbial phyla found in different urban lakes.

Microbial Phyla	Xian, China [38,42]	Nanjing, China [43]	Bandar Sunway, Malaysia [37]	India [29]	Xochimilco Mexico City
*Cyanobacteria*	X	X			
*Proteobacteria*	X	X	X	X	X
*Bacteroidota*		X	X		X
*Firmicutes*		X	X		X
*Actinobacteria*		X	X	X	X
*Chlofoflexi*		X			X

## Data Availability

The original contributions presented in this study are included in the article.
